# Ambulatory anal self-sampling in MSM living with HIV, an acceptable and reliable screening method

**DOI:** 10.1371/journal.pone.0246338

**Published:** 2021-02-09

**Authors:** Benoît Heid-Picard, Béatrix Cochand-Priollet, Flore Rozenberg, David Giang-Phang, Jean-Paul Viard, Valentina La Torre, Jade Ghosn

**Affiliations:** 1 Department of Infectious and Tropical Diseases, Hôpital Bichat-Claude Bernard, Assistance Publique des Hôpitaux de Paris, Université de Paris, Paris, France; 2 Department of Pathology, Hôpital Cochin, Assistance Publique-Hôpitaux de Paris, Université de Paris, Paris, France; 3 Department of Virology, Hôpital Cochin, Assistance Publique-Hôpitaux de Paris, Université de Paris, Paris, France; 4 Departement of Infectious and Tropical Diseases, Hôpital Hôtel-Dieu, Assistance Publique des Hôpitaux de Paris, Université de Paris, Paris, France; 5 Diagnosis and Therapeutic Center, Hôpital Hôtel-Dieu, Assistance Publique-Hôpitaux de Paris, Université de Paris, Paris, France; Baylor College of Medicine, UNITED STATES

## Abstract

**Objectives:**

Anal cancer, usually driven by an oncogenic Human Papillomavirus, remains a leading cause of morbidity in men who have sex with men (MSM) living with HIV, despite combined antiretroviral therapy. Various recommendations advocate to perform regular examination and proctologist-performed samples to anticipate this risk and treat locally before cancer occurrence, an efficient strategy which has the drawback of requiring the proctologist’s availability. This study evaluates the acceptability, feasibility, and efficiency of self-performed samples to screen for HPV-infection and HPV-related anal dysplasia among MSM living with HIV followed in Hôtel-Dieu Hospital.

**Methods:**

Between February 2015 and June 2015, MSM living with HIV and referred to the day-care hospital were offered to perform an anal self-sampling for cytologic and virologic evaluation. A self-sampling kit was provided, and a tutorial video was shown. A subset of participants had a proctology appointment after they did the self-sampling, and thus had a clinical examination and an anal swab sampling performed by the proctologist, using the same sampling material.

**Results:**

Anal self-sampling was offered to 103 patients, and 100 accepted. Sixty-three samples were interpretable, of which 36 (57%) were normal and 27 (43%) showed abnormal results. Virologic analysis was performed for 60 (95%) interpretable samples: 50/60 (83%) of them were positive for HPV. Among HPV-carrier patients, 42/50 (84%) were infected with at least one HR-HPV. Twenty patients had a proctologist consultation. All clinician-performed samples were interpretable and 14 (70%) self-samples were interpretable.

**Conclusions:**

This study highlights the acceptable accuracy of self-sampling screening method among MSM living with HIV and try out its acceptability and feasibility as a secondary prevention device. Although it cannot replace a proctologist consultation for high risk patients, self-sampling should be studied further as one of the ways of screening for anal cancer among low-risk outpatients.

## Introduction

Combined antiretroviral therapy (cART) has dramatically reduced HIV/AIDS-related mortality and morbidity [1]. Anal cancer, however, remains a leading cause of morbidity in men who have sex with men (MSM) living with HIV [2].

In most cases, anal cancer is a squamous cell carcinoma, driven by an oncogenic, or High Risk Human Papillomavirus (HR-HPV), most commonly HPV type 16 and type 18 [3]. Similarly to cervical infection, HPV can cause benign condyloma or may induce intraepithelial anal lesion, the latter displaying a higher prevalence among MSM than among heterosexuals and enhanced by HIV co-infection [4]. Among the general population, HPV is often cleared in 6 months to 2 years. However, among immunocompromised patients, including PLWHIV, annual incidence of high grade squamous intraepithelial lesions is increased, reaching up to 8 to 15% [4].

Despite the use of cART, MSM living with HIV are especially at risk of anal cancer [5], with an incidence of 131 per 100 000 person-years, 30–100 times higher than in the general population, where prevalence ranges from 0.8 to 1.0 per 100 000 persons-year. Among PLWHIV, this association is observed even after immune recovery has been obtained [6], and is not necessarily associated with profound immune deficiency [7].

French guidelines recommend an annual day-care hospitalization to screen for non-communicable, non-AIDS comorbidities in PLWHIV [8]. Currently, there is no consensus about anal dysplasia screening. As this severe complication can easily be prevented by regular anal examination and preventive treatment, French guidelines recommend annual screening for PLWHIV with digital rectal examination and anoscopy, with or without systematic cytology among MSM, patients with condyloma history or women with cervical HPV-related lesions. Systematic screening for viral HPV DNA is not recommended, because the prevalence among MSM living with HIV is around 80% [9].

Yet, the regular examination of an intimate part can raise acceptability issues among PLWHIV [10] and requires an important availability of proctologists’ consultations. These problems can impair the achievement of a systematic screening program and increase its cost. Cytologic anal self-sampling could be an alternative to physician-performed sampling. It has already been studied against different controls and seemed to be acceptable [11–13], performing [14–16] and cost-effective [17].

The purpose of our study was to reassess the acceptability, feasibility, and efficiency of anal self-sampling to screen for HPV-infection and HPV-related anal dysplasia among MSM living with HIV.

## Methods

OVHID is an outpatient cohort of PLWHIV followed in our clinical center. The inclusion in the cohort occurred on the day of admission in the day-care hospital for comprehensive screening of non-communicable, non-AIDS comorbidities according to French guidelines. The OVHID study protocol was approved by the Ethics Committee « Ile de France II » (N° ID RCB: 2010-A00417-32) and by the French general data protection regulation (CNIL). All participants signed written informed consent. MSM living with HIV and referred to the day-care hospital between February 2015 and June 2015 were offered to perform an anal self-sampling. If they were willing to perform such a self-sampling, a short educational video on how to perform the sampling was shown. Patients were advised to introduce the swab for 2 centimeters, to spin it and then to rub the brush against the wall of the preserving vial.

The sampling material was a sterile Dacron swab with a brush dedicated to this kind of anal sampling (Rovers society, Netherlands). The transport medium was a preserving PreserCyt® vial with ThinPrep® liquid, Hologic [18]. The tutorial video used the same material.

All samples were analyzed in the Pathology department of the Cochin Hospital, Paris. Cytologic analyzes were performed using ThinPrep® slides as well as the Papanicolaou staining. The results were reported according to the Bethesda System 2014 used for HPV-linked cervical cancer [19]. All samples with cytologic abnormalities were also tested in the virology department, using the residual ThinPrep liquid, to seek for HPV and its types, using Linear Array genotyping (Roche Laboratory) with MY09-MY11 primers. Samples were classified as satisfactory or unsatisfactory for evaluation following the criteria described in the Bethesda blue book 2014 [19]. To consider a sample adequate it was required to have enough well-preserved nucleated squamous cells; slides with a high number of predominantly anucleate squames or with cells obscured by fecal material or bacteria were considered as inadequate. HPV were classified as HR-HPV (16, 18, 26, 31, 33, 35, 39, 45, 51, 52, 53, 56, 58, 59, 66, 68, 73, 82), Low- Risk HPV (6, 11, 40, 42, 43, 44, 54, 61, 70, 72, 81, CP108) or Undetermined (62, 71, 83, 84, 85, 89) [20].

A subset of participants who had a planned proctology appointment as part of their day-care hospital, because of past anal dysplasia or systematic consultation scheduled every 3 years, did the self-sampling first and then had a clinical examination with high-resolution anoscopy and an anal swab sampling performed by the proctologist, using the same sampling material as described above.

## Results

Anal self-sampling was offered to 103 patients, and 100/103 accepted (97.1%). Their median age was 56 years (min 26, max 75). Median CD4+ count was 704/mm^3^, median CD4+ nadir was 251/mm^3^ (1/mm^3^ to 912/mm^3^). Ninety-four percent of patients had an undetectable plasma viral load and the maximal viral was 550 copies/mL. A history of previous sexually transmitted infections, besides HIV and HPV, was found in 48% of patients, and 19% had a past episode of condyloma.

Out of 100 samples, 63 were interpretable, of which 36 (57%) were normal and 27 (43%) showed abnormal results. Among the samples unsatisfactory for evaluation, 19 (51%) evidenced intestinal glandular cells, 17 (46%) were acellular and 1 (3%) was agglutinated (Table 1). Among the abnormal results, 15 (55%) atypical squamous cells of undetermined significance (ASC-US), 11 (41%) low-grade squamous intra-epithelial lesions (LSIL), and 1 (4%) high-grade squamous intra-epithelial lesions (HSIL) were found (Table 1).

**Table 1:** Cytologic and virologic results of the self-performed samples.

Cytologic Result	Number of patients	Virologic Result	HPV-16	HPV-18	Other HR-HPVs	Low risk	Undetermined risk HPVs
						HPVs (LR-HPV)	
HSIL	1	1/1 (100%) HR-HPV	1/1 (100%)	No	HPV-26 : 1/1	HPV-70 : 1/1	
					HPV-58 : 1/1		
					HPV-82 : 1/1		
LSIL	11	8/11 (73%) HR-HPV	5/11 (45%)	1/11 (9%)	HPV-52: 5/11 (45%)	HPV-6: 3/11 (27%)	HPV-55: 1/11 (9%)
					HPV-33: 2/11 (18%)		
					HPV-56: 2/11 (18%)		
					HPV-66: 2/11 (18%)		
					HPV-31: 1/11 (9%)		
					HPV-39: 1/11 (9%)	HPV-11 : 2/11 (18%)	
					HPV-45: 1/11 (9%)		
					HPV-51: 1/11 (9%)		
					HPV-58: 1/11 (9%)		
					HPV-59: 1/11 (9%)		
		1/11 with undetermined risk or LR-HPV only				HPV- 72 : 1/11 (9%)	1 HPV-62 (among 2/11, 18%)
		1/11 with undetermined risk HPV only					HPV-IS39 : 1/11 (9%)
							1 HPV-62 (among 2/11, 18%)
		1/11 without virologic result					
ASC-US	15	11/15 (73%) HR-HPV	9/15 (60%)	1/15 (7%)	HPV-51: 4/15 (27%)	HPV-11: 2/15 (13%)	HPV-55: 4/15 (27%)
					HPV-52: 4/15 (27%)		
					HPV-31: 3/15 (20%)		
					HPV-45: 1/15 (7%)		
					HPV-53: 1/15 (7%)	HPV- CP6108: 2/15 (13%)	
					HPV-58: 1/15 (7%)	HPV-6: 1/15 (7%)	
					HPV-73: 1/15 (7%)	HPV-61: 1/15 (7%)	
		1/15 with undetermined risk or LR-HPV only				HPV-72: 1/15 (7%)	1 HPV-62 (among 2/15, 13%)
		2/15 with undetermined risk HPV only					HPV-55pof: 1/15 (7%)
							1 HPV-62 (among 2/15, 13%)
		1/15 without virologic result					
Normal	36	22/36 (61%) HR-HPV	7/36 (19%)	3/36 (8%)	HPV-52: 6/36 (17%)	HPV-54: 5/36 (14%)	HPV-62: 7/36 (19%)
					HPV-53: 6/36 (17%)	HPV-61: 4/36 (11%)	
					HPV-51: 4/36 (11%)	HPV-CP6108: 4 (among 5/36, 14%)	HPV-55: 4/36 (11%)
					HPV-56: 4/36 (11%)	HPV-72: 3/36 (8%)	
					HPV-66: 4/36 (11%)	HPV-70: 2/36 (6%)	HPV-69: 1/36 (3%)
					HPV-26: 3/36 (8%)	HPV-42: 2/36 (3%)	
					HPV-31: 3/36 (8%)		HPV-83: 1/36 (3%)
					HPV-33: 3/36 (8%)		
					HPV-35: 3/36 (8%)		HPV-84: 1/36 (3%)
					HPV-59: 3/36 (8%)		
					HPV-45: 2/36 (6%)		
					HPV-58: 2/36 (6%)		
					HPV-68: 2/36 (6%)		
					HPV-73: 2/36 (6%)		
		2/36 (6%) with undetermined risk or LR-HPV only				HPV-11: 1/36 (3%)	
						HPV-54: 1/36 (3%)	
						HPV-81: 1/36 (3%)	
						HPV-CP6108: 1 (among 5/36, 14%)	
		1/36 with undetermined risk HPV only					HPV-83: 1/36 (3%)
		8/36 (22%)					
		Negative PCR					
		3/36 without virologic result					

Virologic analysis was performed for 60 (95%) interpretable samples: 50 (86%) of them were positive for HPV, 8 (13%) were negative, 2 (3%) failed to provide a result (Table 1). Among HPV-carrier patients, 47 (94%) showed a coinfection with several HPV serotypes, 42 (84%) were infected with at least one HR-HPV, 4 (8%) with a low risk HPV, and 4 (8%) with an HPV of undetermined risk without another HPV. Among all samples tested, 22 (38%) were positive for HPV 16, 5 (9%) were positive for HPV 18 (one showing a co-infection with HPV16). Besides, 15 (26%) evidenced HPV 52, 9 (16%) HPV 51, 7 (12%) HPV 31, 7 (12%) HPV 53, 6 (10%) HPV 56, 6 (10%) HPV 66, 5 (9%) HPV 33, 5 (9%) HPV 58, 4 (7%) HPV 26, 4 (7%) HPV 59, 4 (7%) HPV 45, 1 (2%) HPV 39, 3 (5%) HPV 35, 3 (5%) HPV 73, 2 (3%) HPV 68, and 1 (2%) HPV 82 (Table 2). A detailed overview of the correspondence between cytologic and virologic results is provided in Table 2.

**Table 2:** Comparison of self-performed and clinician-performed sampling.

Self-performed Sampling Result	Number of patients (%)	Clinician-performed Sampling Result
Unsatisfactory samples : 6	6 (30%)	Satisfactory samples
		Normal
Satisfactory sample	1 (5%)	Satisfactory sample
1 HSIL		LSIL
Satisfactory samples	3 (15%)	Satisfactory samples
		LSIL
4 LSIL	1 (5%)	Satisfactory sample
		Normal
Satisfactory samples	1 (5%)	Satisfactory sample
		ASC-US
5 ASC-US	1 (5%)	Biopsy performed:
		Condyloma and LSIL
	3 (15%)	Satisfactory samples
		Normal
Satisfactory samples	2 (10%)	Satisfactory samples
		Normal
4 Normal	2 (10%)	Satisfactory samples
		ASC-US

No significant differences were found between patients with cytological lesions and patients with normal self-sample except the CD4+ count, respectively at 690 /mm^3^ (227–1476) and 865/mm^3^ (164–1760) (Student’s Test p<0.05).

Twenty patients had an examination with a proctologist consultation planned for their day hospitalization, and thus had both self-sample and clinician-performed samples on the same day (3 of them had a biopsy instead of a sample, because of macroscopic lesions). All clinician-performed samples were interpretable. Fourteen (70%) self-samples were interpretable and 6 (30%) were not (Table 2). Among the patients with uninterpretable self-sample, all clinician-performed sample were normal. Self-samples evidenced 5 ASC-US (1 ASC-US, 1 LSIL with condyloma and 3 normal samples in the corresponding clinician-performed samples), 4 LSIL (3 LSIL and 1 normal sample in the clinician-performed samples), 1 HSIL (LSIL in the clinician-performed sample) and 4 normal samples (2 normal samples and 2 ASC-US in clinician-performed samples) (Table 2).

The 3 biopsies evidenced 1 low-grade dysplasia (self-sample showed LSIL), 1 low-grade dysplasia with condyloma (self-sample showed ASC-US) and 1 normal biopsy (self-sample showed ASC-US).

Only 1 patient had an HSIL according to the clinician-performed sample. Self-sample concluded to LSIL without being able to exclude HSIL and advised to perform a biopsy. The virologic sample evidenced HPV 16, HPV 26, HPV 58, HPV 70 and HPV 82.

## Discussion

According to the studies we reviewed, the prevalence of histologically-proven squamous intra epithelial neoplasia in MSM living with HIV is around 20% [21] which is quite similar to cervical cancer before the development of screening programs for women [22].French guidelines recommend annual screening for MSM living with HIV, with digital rectal examination and anoscopy, with or without systematic cytology [8]. Yet, outpatients with uncomplicated, well controlled HIV infection are numerous and merely followed with consultations and annual day hospitalization. Their screening would require an important number of examinations by proctologists, who are not available at every care center. Patient-performed self-samples could be a cost-efficient method for screening this population of outpatients [26]. Self-sampling for screening of cervical cancer in women has already proven its feasibility [23, 24] and cost efficiency [25], and virologic tests keep being developed at lower costs [26]. Among MSM, anal self-sampling has already been studied against different controls and seemed to be acceptable [11–13], performing [14–16] and cost-effective [17].

Hereby, in a cohort of 100 patients, followed according to French guidelines with consultations and day hospitalization, we found a strong acceptability (97%), like in previous studies concerning PLWHIV [11, 13, 14], and a satisfactory accuracy (63%) of anal self-sampling. Among patients with interpretable samples, 86% were positive for HPV, of whose 84% were infected with a high-risk HPV. These numbers are close to those found in similar studies [13, 14, 27], and the variations of accuracy between cohorts could be attributed to different technical conditions and definitions of interpretability. On the counterpart, only 63% of the self-samples have been considered as “satisfactory for evaluation” which is a close percentage than in other studies [14, 27]. Our criteria to consider a sample “satisfactory for evaluation” were strict as previously explained. The criteria of adequacy and non-adequacy are well described and applied for the cervical cytology but there is a paucity of references concerning the anal cytology. In the study with the best results in terms of adequacy for anal self-sampling i.e. around 80% of adequacy [16], the criteria for adequacy were not described; it was only specified that “the number of nucleated squamous cells was sufficient or not” without any further comments. One of the points of our study was to compare the effectiveness of clinician-collected versus self-collected samples. 20% of patients had a consultation with a proctologist and a clinician-collected sample. The proportion of interpretable samples (100%) was greater than for self-samples (63%). There are some discrepancies between self- and clinician performed samples. We could expect the clinician-performed ones to be more accurate to find higher grade lesions, being sampled with direct vision and with our proctologists’ experience. We evidenced higher grade lesions with self-sampling for 5 (25%) patients, with clinical performed for 3(15%) patients, and concordant results for 6 (30%) patients. In this small number of patients, a higher performance to detect higher grade lesions wasn’t evidenced. The number of patients with a proctologist-performed sample was limited, which reduces the strength of the comparison, but we think that self-sampling, although less efficient, could be an acceptable first-line method for screening. Uninterpretable or anormal self-sampling should then lead to perform a proctologist consultation with anoscopy.

This study reports a prospective systematic screening cohort of 100 ambulatory patients, with cytologic and virologic results of self-samples for most of the patients. Despite being single-centered, it provides a viewing of the prevalence of AINs among MSM living with HIV. It corroborates the previously obtained data studies, concerning an important topic for MSM living with HIV which lacks cost-efficient screening methods. Furthermore, it provides an overview of HPV carriage among MSM living with HPV. This preliminary study does not aim to establish anal self-sampling as substitute for proctologist consultation. However, it does highlight the acceptable accuracy of this screening method among MSM living with HIV and try out anew its acceptability and feasibility as a secondary prevention device. Besides, considering the cost of screening and treating HPV infection, investigations for the efficiency of the HPV vaccine among MSM living with HIV. Just as it was recently investigated among women living with HIV [28], it should be assessed in MSM, especially in young MSM, in parallel to the assessment of effective screening methods to prevent from anal cancer in those already harboring HR-HPV. This work provides an interesting overview of HPV-carriage epidemiology among MSM living with in HIV and followed in Paris. We found an important carriage of HR-HPV, mainly HPV-16, HPV-52, HPV-51, HPV-31, HPV-56 and HPV-66. Some of these strains are currently not include in the marketed vaccines (Gardasil®, Gardasil9® and Cervarix®), but a cross-protection after HPV-vaccination has already been suggested [29]. Further studies are needed to evaluate the benefit of adapting the vaccine-strains to the different group of individuals, mainly based on their sexual activity habits [30].

To conclude, considering the high prevalence of HR-HPV among MSM living with HIV and the satisfactory accuracy of this low-priced ambulatory test, self-sampling strategy should be studied further as one of the ways of screening for anal cancer, among others. Further prospective studies are needed to evaluate its use among PLWHIV and to distinguish the respective usefulness of cytologic and virologic samples. Caution should be taken for high-risk patients, for instance those with history of condyloma, before skipping the visit with the proctologist. Higher performance of a clinician-performed sample and absence of direct visual control during a self-sample should be kept in mind. Yet, acceptability and proctologist availability being the main barriers to the consultation, positive anal sampling could help to convince the patient to attend proctologist’s consultation and should help to target the higher risk MSM living with HIV.
